# Evaluation of the Effect of Benzydamine Hydrochloride on the Intensity of Gag Reflex: A Randomized Single-Blind Clinical Trial

**DOI:** 10.30476/dentjods.2023.97675.2032

**Published:** 2024-06-01

**Authors:** Mohammad Mehdi Torabi, Reyhaneh Shoorgashti, Farnaz Haji Fattahi, Simin Lesan

**Affiliations:** 1 Dentist, Tehran, Iran; 2 Dept. of Oral Medicine, Islamic Azad University of Medical Sciences, Dental Branch, Tehran, Iran

**Keywords:** Reflex, Gagging, Benzydamine, Local anesthetic, Lidocaine

## Abstract

**Statement of the Problem:**

Gag reflex is among the most common problems during dental and endoscopic procedures. Benzydamine hydrochloride is a non-steroidal anti-inflammatory medication and a local anesthetic that might be useful in reducing the gag reflex.

**Purpose:**

This study aimed to evaluate the effects of benzydamine hydrochloride mouthwash on the intensity of the gag reflex.

**Materials and Method:**

In this randomized clinical trial study, 30 participants aged 21-26 with a gag trigger point index (GTPI) higher than 2 were divided into 2 groups. In the case group, 15 ml of 0.15% benzydamine hydrochloride mouthwash was gargled for 1 minute, and after 10 minutes GTPI test was conducted. In the control group, 4 puffs of 10% lidocaine spray were applied to the mucosa of the targeted area, and after 5 minutes, GTPI was measured. Participants were asked about the taste and smell of the medications.

**Results:**

GTPI was significantly reduced in both groups. In the lidocaine group, the GTPI score changed from 4.47 to 2.00 (*p*< 0.001), and that for the benzydamine group was 4.20 to 1.47 (*p*< 0.001). The variance rate of the gag reflex was -2.73 and -2.47 in the benzydamine group and lidocaine group, respectively. However, this reduction was not statistically significant between the two groups. Moreover, benzydamine mouthwash has a significantly better taste and smell than lidocaine (*p*= 0.001).

**Conclusion:**

The results of this study showed that benzydamine mouthwash could be used quite effectively in reducing the gag reflex.

## Introduction

The gag reflex is one of the defensive mechanisms that protect respiratory tracts from foreign objects’ aspiration. The intensity and stimulators of this reflex can be varied among individuals [ 1 - 3 ]. During dental and endoscopic procedures, the gag reflex is a common problem [ 4 - 7 ]. Studies show that 8.2% of dental patients experience this issue. Moreover, during denture fitting sessions, the incidence of gagging is higher, with 44% of patients reporting it [ 8 - 10 ]. Having an overactive gag reflex can be upsetting for patients and may result in them avoiding regular dental appointments, neglecting oral hygiene, and even losing teeth. It is reported that gagging-related issues account for 20% of dental avoidance cases [ 8 ].

Benzydamine hydrochloride is known as an anti-inflammatory medication. This non-steroid drug can cause local anesthesia and concentrate in infectious sites. So that, its systemic side effects can be neglectable, resulting in a competitive advantage over other painkillers like non-steroidal anti-inflammatory drugs (NSAIDs) [ 11 - 12 ]. Mouthwashes containing this component are commonly used to manage the pain of oral aphthous, periodontal surgeries, oral mucositis, and postintubation sore throat. The benzydamine hydrochloride mechanism for pain reduction is sodium channel blockage, similar to other local analgesic medications such as lidocaine [ 11 , 13 - 15 ]. 

There have been multiple proposed techniques to manage the gag reflex, but regrettably, no definitive solution has been found yet. The importance of discovering a reliable method to control the gag reflex has become more critical during the COVID-19 outbreak. This is because the virus is present in the saliva of infected persons, and practitioners face a higher risk of infection due to increased saliva secretion when gagging occurs. Consequently, it is essential to allocate funding toward finding a guaranteed way to manage the gag reflex [ 16 ].

To date, there has not been any research conducted on the impact of benzydamine hydrochloride on the gag reflex. Solutions like acupuncture and acupressure using thumb pressure with or without sedation showed uncertain evidence regarding the successful reduction in gaging. Prescribing medications like lidocaine is often unbearable for patients due to its bitter taste, and there have been some cases of allergic reactions, and overdoses reported [ 1 , 17 - 21 ]. As a result, it is imperative to explore new options. The aim of this study is to evaluate the effect of benzydamine hydrochloride on the gag reflex.

## Materials and Method

The methods of this clinical trial were approved by the ethical community under the ethical code of IR.IAU. DENTAL.REC.1399.069, and its Iranian registry of clinical trials (IRCT) is IRCT20190608043843N2.

Consolidated standards of reporting trials (CONSORT) 2010 was used to report this randomized, single-blind, and parallel study. The sample size of this study was calculated as 15 subjects in each group by means of Paired Means Power Analysis option in SPSS22 and considering α=0.5, β=0.2, and the effect size= 0.6.50 individuals who volunteered to participate in the study were evaluated regarding the inclusive and exclusive criteria, which resulted in the selection of 30 participants out of 50. 

30 selected participants (15 women and 15 men) were randomly divided into 2 groups using the method of block randomization: 1. Benzydamine hydrochloride mouthwash 2. Lidocaine spray (Control group). We divided the participants into 15 blocks, with randomly assigned treatment and control orders. Participants were also randomly assigned to each block, and the treatment order was randomized within each one.

After obtaining informed consent, the intensity of the gag reflex in all volunteers was measured using the gag trigger point index (GTPI), and their score was recorded on a survey accounted for each person [ 22 ]. Volunteers who scored higher than 2 were included in the study, and participants who were pregnant or in the breastfeeding period, suffered from any systemic diseases, motor neuron disease, or had an allergy to benzydamine hydrochloride or lidocaine were excluded from the study. The participants selected had similar demographic characteristics, as they were all students of the same university, Islamic Azad University, dental branch, Tehran, Iran, between the ages of 21 and 26 [ 22 - 23 ]. In addition, all examination was conducted at the Oral Medicine Department of this university. 

All volunteers were examined between 9 and 11 a.m. and asked to eat breakfast 2 hours before the examination. Disposable wooden tongue blade was used to stimuli areas mentioned in Table 1. The tongue blade touched the oral mucosa from the anterior to the posterior areas. In order to reduce the mental interference factors, no information about the methodology was given to participants. The drug application and the gag measurement were performed for each participant separately and apart from the rest. All examinations were applied by a trained dental student under the supervision of an oral and maxillofacial medicine expert at the up- right position on a specific dental unit at Oral Medicine Department of Islamic Azad University, dental branch, Tehran, Iran. The selection, recording, and applying the medication were done by another dental student.

**Table 1:** A description of the gag trigger point index (GTPI) score coded by the location in the oral cavity where the gag reflex occurs [ 22 ]

Location of gag trigger point	GTPI* Score
Posterior pharyngeal wall, No motor response	0
Posterior pharyngeal wall, Motor response	1
Between posterior faucial pillars and posterior pharyngeal wall	2
Posterior faucial pillars	3
Between anterior faucial pillars and posterior faucial pillars	4
Anterior faucial pillars	5
Between second molars and anterior faucial pillars	6
Second Molars	7
Internal cheek, Center	8

* Gag Trigger Point Index

Benzydamine hydrochloride was poured into the same bottle as lidocaine spray, and both bottles were completely covered with black-colored electrical tape. This was done to ensure that participants did not make any assumptions about differences between the two medications before their application and kept them blind. In the benzydamine hydrochloride mouthwash group, 15 milliliters (mL) of benzydamine hydrochloride mouthwash 0.15% (Behvazan company: Iran) was gargled for 1 minute, and after 10 minutes GTPI test was done exactly with the same approach, applied before the intervention [ 24 ]. In the lidocaine group, 4 puffs of the spray of lidocaine 10% (Iran Darou company: Iran) were used, and the GTPI test was done after 5 minutes [ 25 ]. 

For each participant, the test was done on only one side (right or left) before and after the intervention. Patient satisfaction was assessed through a qualitative self-report survey. Patients were given the option to choose from "good”, "moderate," or "weak" ratings regarding the taste and smell of the medication they were using. 

The data were collected using a survey, observation, and clinical examination and analyzed using the Wilcoxon sign rank test in SPSS22. The level of significance was considered 0.05. 

## Results

In this study, there was no significant difference between the two groups regarding demographic features. All participants were studying at the same university. All of them were aged between 21 to 26 years old, and the number of men and women participants was equal (15 women and 15 men) (*p*< 0.3) (Table 2) [ 22 ].

**Table 2:** The features of the studied participants in terms of gender and age

Groups (n.)		Benzydamine hydrochlorid (15)	Lidocaine spray (15)	Wilcoxon sign rank test result
Variables				
Mean Age		23.6	22.73	0.250
Gender	Men	9	8	0.713
	Women	6	7	

**Figure 1:**
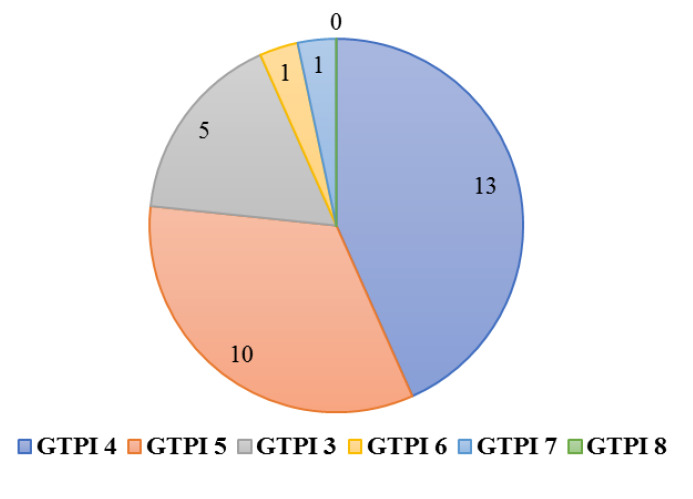
The pie chart of the frequency of participants in each gag trigger point index (GTPI) score

**Figure 2:**
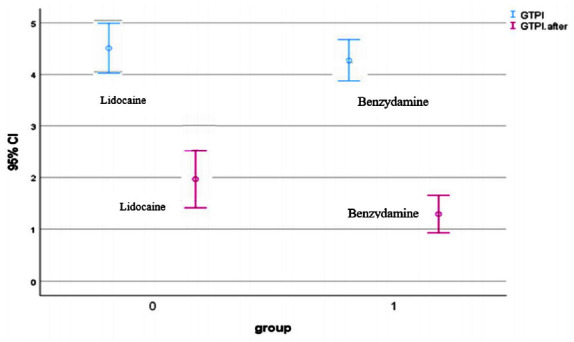
Error bar chart showing the changes in the mean gag reflex in two groups before and after the intervention

In terms of GTPI test conducted before the intervention, 13 participants scored 4, 10 participants scored 5, 5 participants scored 3, 1 participant scored 6, and 1 participant scored 7 (Figure 1). In addition, 17 volunteers showed a GTPI score of less than 2, resulting in exclusion from the study (Figure 2).

Table 3 shows the intensity of the gag reflex among the participants before and after the intervention. There was a negligible difference between the 2 groups regarding this intensity before the intervention (*p*= 0.512).

**Table 3:** The mean, SD, and *P* value of the GTPI score of the examined participants in each group

Groups	The lowest GTPI score	The highest GTPI score	Mean±SD	Variance range	*p* Value
Benzydamine hydrochloride (Before the intervention)	2	6	4.20±0.862	-2.73± 0.263	<0.001
Benzydamine hydrochloride (After the intervention)	0	4	1.47±1.125		
Lidocaine spray (Before the intervention)	3	7	4.47±0.990	-2.47± 0.205	<0.001
Lidocaine spray (After the intervention)	0	4	2.00±1.195		

Participants of both groups showed a reduction in GTPI score after taking medications, and based on the Wilcoxon sign rank, the range of variance was significantly different in each group (*p*< 0.001). However, this range was not considered between the 2 groups (*p*< 0.025) (Table 3).

Regarding the patient satisfaction with the taste and smell of the 2 medications, a significant difference was seen in the 2 groups, and the participants were more satisfied with the taste and smell of benzydamine hydrochloride mouthwash rather than lidocaine spry (*p*= 0.001) (Tables 4-5).

**Table 4:** The level of satisfaction of the participants regarding the taste of the medications

Drug taste	Good	Moderate	Weak	*p* Value
Groups				
Benzydamine hydrochloride	11	3	1	0.001
Lidocaine spray	0	2	13	

**Table 5:** The level of satisfaction of the participants regarding the smell of the medications

Drug smell	Good	Moderate	Weak	*p* Value
Groups				
Benzydamine hydrochloride	11	3	1	0.001
Lidocaine spray	0	7	8	

## Discussion

The results of this study revealed that both benzydamine hydrochloride mouthwash and lidocaine spray can reduce the intensity of gag reflex in patients, and there is no significant difference between using these 2 medications. However, patients may prefer benzydamine hydrochloride because of its better taste and smell. While the lidocaine spray is more tolerable and satisfying in comparison to its solution or mouthwash [ 21 ], compared to benzydamine hydrochloride, it has not decent-taste and smell. In this study, over 70% of participants who used benzydamine hydrochloride described its taste and smell as good, while none in the lidocaine group reported this.

The gag reflex can be a huge problem when it comes to dental or endoscopic procedures. The prevalence of this problem is considered to be 8.2 to 44% among patients needing dental treatments [ 4 - 7 ]. This reflex is mainly triggered by five regions in the oral cavity, which include the base of the tongue, the fauces, the palate, the uvula, and the posterior wall of the pharynx. Medulla oblongata receives afferent fibers from the trigeminal, glossopharyngeal, and vagus nerves when stimulated intraorally [ 1 , 26 ]. Different approaches were suggested to reduce the intensity of gagging, including prescribing medications and non-pharmacological methods like distraction techniques, acupuncture, acupressure, and relaxation techniques [ 27 - 30 ]. Using local anesthetics as a solution for gagging problems dates back to 1977 when Kramer and Braham [ 31 ] raised the hypothesis that patients will be less likely to gag if the mucosal surfaces of the soft palate are desensitized. Desensitization of the oral tissues can be accomplished using spray, gel, lozenge, mouthwash, or injection of local anesthetics [ 1 ]. Lidocaine and benzydamine hydrochloride are two types of these anesthetics [ 20 , 32 - 33 ]. 

Benzydamine is an anti-inflammatory component that can be used for its anesthetic and analgesic effects. Its hydrochloride salts are used systemically and locally in the fields of odontostomatology, otorhinolaryngology, orthopedics, urology, and gynecology [ 34 ]. Mucositis caused due to radiotherapy, and sore throats can both be reduced with benzydamine hydrochloride, which has been used for decades in patients undergoing general anesthesia [ 11 - 12 , 14 , 25 ]. The effects of this drug on gagging have yet to be studied, and this study is the first evaluation of the relationship between gargling benzydamine hydrochloride and the intensity of the gag reflex.

As a result of gargling, the component of the mouthwash can be effectively distributed into the area of the oropharynx, posterior pharynx, epiglottis, and uvula. It is found that if benzydamine hydrochloride is present at concentrations between 10 and 100 micromol/ litter, the mucous membrane may be stabilized [ 11 ]. Beta-blockers and some acidic non-steroidal anti-inflammatory drugs (NSAIDs) have a similar mechanism to benzydamine hydrochloride. They can eliminate the secretion of azurophilic granules from the innate immune cells. It also can lead to a growth in the production of cyclic 3'-5' adenosine monophosphate (cyclic3′-5′-AMP), by which the intracellular cation activity can be affected [ 11 ]. Besides, benzydamine can block sodium channels expressed in the sensory nerves and reduce the stimulation of nociceptors related to capsaicin. All these activities lead to benzydamine hydrochloride's anesthetic and anti-inflammatory properties [ 32 ].

Lidocaine is widely used for the reduction of pain and gag reflex in previous studies, and its efficiency has been approved [ 21 , 33 , 35 - 37 ]. However, its side effects can be considerably more than benzydamine mouthwash. It appears that using lidocaine spray on the oral pharyngeal cavity prior to intubation can lead to a higher frequency and severity of postoperative sore throat [ 38 ]. Using 10% lidocaine could result in damage to the mucosa due to its solvent additives. These additives, like menthol and ethanol, can irritate the mucosa [ 38 ]. Nausea, vomiting, and dysphagia can be the other possible side effects of lidocaine spray [ 11 ]. 

Local anesthetic systemic toxicity (LAST) can be a serious and life-threatening condition that affects both the central nervous system and cardiovascular system [ 39 ]. Chen *et al*. [ 40 ] reported that LAST might occur through different methods of lidocaine administration, including local sprays. Early warning signs and symptoms of LAST typically involve the central nervous system and frequently observed. Although rare, there have been reported cases of quadriparesis following the administration of lidocaine [ 39 - 40 ]. Our study found no side effects for benzydamine mouthwash, but Romita *et al*. [ 34 ] reported two cases of photoallergic contact cheilitis caused due to benzydamine hydrochloride mouthwash in 2020.

Although no difference was seen between lidocaine and benzydamine hydrochloride in our study, Mekhemar *et al*. [ 38 ] showed that benzydamine has better performance than lidocaine regarding the reduction of sore throat after tracheal extubation. Other than that, the analgesic effects of benzydamine mouthwash last significantly longer than those of lidocaine spray. In a conducted study by Sugiarto *et al*. [ 11 ], it was shown that the effects of topical anesthesia caused as a result of benzydamine could start after 30 seconds of gargling 4 millimole/ litter in 15 milliliters (mL) and lasts up to 90 minutes; but when lidocaine spray is applied to the oral mucosa, its analgesic effect lasts for only 15 minutes [ 11 , 38 ].

The limitation of this study was an impossible approach to conduct it as a double-blinded clinical trial study due to the different forms of two drugs (spray and mouthwash). In addition, more studies are recommended to investigate the duration of the effect of benzydamine in different dental procedures.

## Conclusion

The Results of this study showed that benzydamine hydrochloride mouthwash could be used quite effectively without side effects in local anesthesia and reduction of the gag reflex. The use of benzydamine mouthwash significantly reduced the rate of gag reflex, similar to lidocaine spray in this study. Because of this drug's better taste and smell and fewer side effects compared to lidocaine, this medication can be a suitable alternative to lidocaine. 
